# Comparison between Symptomatic and Asymptomatic Mice after *Clostridioides difficile* Infection Reveals Novel Inflammatory Pathways and Contributing Microbiota

**DOI:** 10.3390/microorganisms10122380

**Published:** 2022-11-30

**Authors:** Ahmed AbdelKhalek, Sanjeev K. Narayanan

**Affiliations:** 1 Department of Comparative Pathobiology, Purdue University, 625 Harrison Street, West Lafayette, IN 47907, USA; 2 Purdue Institute of Inflammation, Immunology, and Infectious Disease, West Lafayette, IN 47907, USA

**Keywords:** *Clostridioides difficile*, mouse model, inflammation, *OSM*, *MMP*, *Trem-1*, *Duox2*, microbiome, *Turicibacter*, *Citrobacter amalonaticus*

## Abstract

*Clostridioides difficile* causes the highest number of nosocomial infections. Currently, treatment options for *C. difficile* infection (CDI) are very limited, resulting in poor treatment outcomes and high recurrence rates. Although the disease caused by CDI is inflammatory in nature, the role of inflammation in the development of CDI symptoms is contradictory and not completely understood. Hence, the use of anti-inflammatory medication is debatable in CDI. In the current study, we evaluated the genetic and microbiome profiles of mice after infection with *C. difficile*. These mice were categorized based on the severity of CDI and the results were viewed accordingly. Our results indicate that certain genes are upregulated in severe CDI more than in the moderate case. These include oncostatin-M *(OSM),* matrix metalloprotease 8 *(MMP8),* triggering receptor expressed on myeloid cells 1 *(Trem-1*), and dual oxidase 2 *(Duox2)*. We also investigated the microbiome composition of CDI mice before and after infecting with *C. difficile*. The results show that *C. difficile* abundance is not indicative of diseases severity. Certain bacterial species (e.g., *Citrobacter*) were enriched while others (e.g., *Turicibacter*) were absent in severe CDI. This study identifies novel inflammatory pathways and bacterial species with a potential role in determining the severity of CDI.

## 1. Introduction

*Clostridioides difficile* (previously known as *Clostridium difficile*) is an anaerobic Gram-positive bacterium that causes severe and, in some cases, fatal diarrhea. Since the year 2000, *Clostridioides difficile* infection (CDI) has been regarded as one of the most dangerous hospital-acquired infections [1,2]. The number of CDI treatment options is very limited and the rate of treatment failure and recurrence after clinical resolution following the use of the current medications is high [3]. Accordingly, the Centers for Disease Control and Prevention (CDC) listed CDI as an urgent threat that required immediate development of new therapeutics [1]. *C. difficile* persists in the environment by means of its dormant spores that are resistant to oxygen, heat, and chemicals. Presence of *C. difficile* spores in the intestine of susceptible individuals, from an endogenous source, or after fecal–oral contamination, initiates CDI [4,5,6]. *C. difficile* spores germinate in the intestine upon exposure to bile salts to form toxin-producing vegetative cells. Disturbance of the normal intestinal bacterial flora (due to the administration of broad-spectrum antibiotics, for example), old age, hospitalization, and comorbidities (e.g., HIV/AIDS and cancer) are the major risk factors for CDI [3,7,8]. There is also a concern regarding the potential increase in CDI cases as a result of the current coronavirus (COVID-19) pandemic. The difficulty of applying regular preventative measures, increased antibiotic usage and reduced testing for CDI during COVID-19 pandemic can all result in a surge of CDI incidence and severity [9,10,11].

Virulent strains of *C. difficile* produce two major enterotoxins, TcdA and TcdB [12,13]. Both synergistically inactivate cellular small GTPases after entry into the host cell. This results in disruption of the actin cytoskeleton, loss of epithelial tight junctions, increased permeability, and cell death (cytopathic effect). In addition, toxins trigger the inflammasome and activate nuclear factor-κB (NF-κB) pathways with the release of proinflammatory cytokines and chemokines which leads to an inflammatory state with the recruitment of neutrophils (cytotoxic/enterotoxic effect) [12,13,14]. Neutrophil infiltration is a hallmark of CDI, contributing to mucosal inflammation and damage. *C. difficile*-induced inflammation has an uncertain role in CDI progression, and it is not clear whether it is beneficial or damaging to the host. Immune pathways thought to have beneficial effects include fractalkine (CX_3_C), innate immune sensors (toll-like receptor 5 [TLR5], and nucleotide-binding oligomerization domain 1 [NOD1]), and neutralizing antibodies. Immune reactions such as neutrophil infiltration and the release of interferon gamma (IFN-γ), tumor necrosis factor alpha (TNF-α), leptin, interleukin 8 (IL-8), and substance P are believed to be harmful to the host [12,13,15,16]. Recently, *C. difficile* was shown to benefit from the inflammatory state of the host intestine. Fletcher et al. demonstrated the metabolic activities (metabolome) of *C. difficile* to be different when the host intestine is in an inflammatory state. Inflammation upregulates the expression of matrix metalloproteinases (*MMPs*), which breaks down collagen in the extracellular matrix releasing proline and other amino acids that can be directly utilized by *C. difficile* via Stickland reaction. In addition, the inflammatory state of the host excluded protective members of the normal gut flora [17].

In most CDI mouse experiments performed by others and us, there is a subset of infected mice that do not develop even a mild disease [18,19,20,21,22]. Since the mice used are inbred (genotypically similar) and co-habitated (similar, but not identical gut microflora), the exact cause for the difference in disease severity and progression between infected symptomatic and infected asymptomatic mice is not well-understood. Identifying the differences in the host gene expression (transcriptome) and gut microbiome between uninfected mice, infected mice with symptoms typical to CDI, and infected mice with no CDI symptoms will allow us to identify novel factors influencing *C. difficile* pathogenesis. 

*The current study is devised to compare the host factors and gut bacterial flora associated with either severe or asymptomatic CDI.* We utilized a mouse infection model of CDI and used RNA-seq and microbiome analysis to compare the profiles of uninfected and infected symptomatic and asymptomatic mice. Out of 1812 upregulated genes in mice with CDI, we selected the most upregulated and inflammatory-related genes to study further. We also investigated the difference in microbiome composition between symptomatic and asymptomatic mice before and after infection with *C. difficile*, and evaluated their intestinal histology. 

Identification of novel contributors to *C. difficile*-induced inflammation that can be modulated could define treatment approaches to improve the outcome of CDI. This modulation can be a stand-alone or an add-on strategy to the current treatment regimens.

## 2. Materials and Methods

### 2.1. Chemicals and Bacterial Strains

*C. difficile* strain ATCC 43255 was obtained from Microbiologics (St. Cloud, MN, USA). *C. difficile* ATCC 43255 (VPI 10463) is a ribotype 087, toxigenic strain that produces both toxins A and B. This strain is commonly used in mouse infection models of CDI. Vancomycin hydrochloride (Gold Biotechnology, St. Louis, MO, USA), metronidazole (Beantown Chemical Corporation, Hudson, NH, USA), kanamycin, gentamycin, and colistin (TCI America, Portland, OR, USA) were procured from commercial vendors. Brain heart infusion (BHI) was purchased from Becton, Dickinson, and Company (Cockeysville, MD, USA). Phosphate-buffered saline (PBS), fetal bovine serum, and non-essential amino acids (NEAA) were purchased from Fisher Scientific (Waltham, MA, USA). Yeast extract, L-cysteine, vitamin K, and hemin were obtained from Sigma-Aldrich (St. Louis, MO, USA). The RNA extraction kit (RNeasy^®^ Mini), DNase, and reverse transcription kit (QuantiTect^®^) were purchased from Qiagen (Germantown, MD, USA). PowerUp™ SYBR™ Green Master Mix (Applied Biosystems, Waltham, MA, USA) was purchased from Fisher Scientific. 

### 2.2. Mouse Model of C. difficile Infection

Mouse studies were in accordance with the American Veterinary Medical Association (AVMA) guidelines and were approved by Purdue Institutional Animal Care and Use Committee (IACUC) under protocol number 2008002068. Antibiotic-primed mouse model of CDI was utilized as described previously [23,24]. Briefly, six-week-old C57BL/6 mice (n = 6, Jackson laboratory, Bar Harbor, ME, USA) where acclimatized for one week before oral treatment with five-antibiotic cocktail (kanamycin [0.4 mg/mL], gentamicin [0.035 mg/mL], vancomycin [0.045 mg/mL], metronidazole [0.215 mg/mL], and colistin [850 U/mL]) for three days. Antibiotic treatment was then ceased for two days to allow for the drug clearance before injecting the mice intraperitoneally with clindamycin (10 mg/kg). One day later, mice were orally infected with 5 × 10^5^ CFU/mL *C. difficile* ATCC 43255 spores. Uninfected mice were used as a control. Mice were monitored for signs of CDI (including diarrhea, scuffed coat, hunching, inability to eat or drink, lethargy, and unresponsiveness) and were euthanized immediately upon the appearance of severe signs. Untreated and asymptomatic mice were euthanized at the end of the experiment using CO_2_ asphyxiation and organs were collected for histopathological examination. Fecal samples were collected at three time points during the experiment, before antibiotic administration, before spore inoculation, and right after euthanasia (colon content).

### 2.3. RNA Purification and Transcriptomic Analysis

RNA was extracted from the cecal mucosa and cecal contents of mice upon euthanasia using RNeasy extraction kit (Qiagen) as per manufacturer’s instructions. Residual DNA was removed using on-column digestion with RNase-free DNase (Qiagen). One part of the extracted RNA was sent to Novogene (Sacramento, CA, USA) to perform RNA-seq analysis for one mouse from each group. The second part of RNA was used to synthesize complementary DNA (cDNA) utilizing QuantiTect^®^ Reverse Transcription Kit (Qiagen). cDNA was used to detect differential expression of selected genes (*Trem-1*, *OSM*, *Duox2,* and *GAPDH* [house-keeping gene]) via quantitative PCR (Q-PCR). Q-PCR primers were designed using the Primer3web online tool and synthesized by Integrated DNA Technologies (IDT, Coralville, IA, USA). Each 10 µL Q-PCR reaction consisted of 5 µL PowerUp™ SYBR™ Green Master Mix, 500 nM of each of the forward and reverse primers, and 5 ng of cDNA. Q-PCR was performed by QuantStudio 5 real-time PCR system (Applied Biosystems) and Ct values were calculated using QuantStudio Design and Analysis software v1.5.1. Thermal cycles consisted of Uracil-DNA Glycosylase activation at 50 °C for 2 min, initial denaturation at 95 °C for 2 min, followed by 40 cycles of denaturation (95 °C for 15 s), annealing (53–55 °C for 15 s), and extension (72 °C for 60 s). A single melt-curve peak temperature (Tm) was observed for each PCR reaction.

### 2.4. Histopathological Analysis of Mice Tissues

Mice intestines were collected right after euthanasia, formalin-fixed, and transferred on the next day to 70% ethanol. Tissues were embedded in paraffin, sectioned, and stained with hematoxylin and eosin (H&E) for histopathological examination. Tissue processing was performed by the Histology Research Laboratory at Purdue University. 

### 2.5. Difference in Microbiome Composition of Infected Symptomatic and Infected Asymptomatic Mice

Microbiome analysis was performed by TransnetYX (Cordova, TN, USA). Infected mice were classified according to the severity of CDI symptoms to severe (mice showed exaggerated symptoms of weight loss [>20%], diarrhea, hunched posture, and death or had to be euthanized immediately), moderate (mice showed mild symptoms and survived till the end of the experiment), and asymptomatic mice. Fecal pellets and the colon content of one mouse from each group were collected at three timepoints: before the start of the antibiotic cocktail, after antibiotic administration (immediately before infection), and after euthanasia (colon contents). DNA extraction was performed via the DNeasy 96 PowerSoil Pro QIAcube HT extraction kit (Qiagen) following the manufacturer’s protocol. This process ensures reproducible extraction of inhibitor-free, high-molecular-weight, genomic DNA that captures the true microbial diversity of stool samples. After DNA extraction and quality control (QC), genomic DNA was converted into sequencing libraries (KAPA HyperPlus library preparation protocol) and unique dual indexed (UDI) adapters were used to ensure that reads and/or organisms are not mis-assigned. After QC, the libraries were sequenced using a shotgun sequencing method (Illumina NovaSeq, at a depth of 2 million 2 × 150 bp read pairs), which enables species- and strain-level taxonomic resolution. Sequencing data were uploaded automatically onto One Codex analysis software and analyzed against the One Codex database consisting of approximately 115,000 whole microbial reference genomes (including 62,000 distinct bacterial genomes, 48,000 viral genomes, and thousands of archaeal and eukaryotic genomes). 

### 2.6. Statistical Analysis

GraphPad software version 7.0 (GraphPad Software, La Jolla, CA, USA) was used for graph design and statistical analysis. One-way analysis of variance (ANOVA) followed by Tukey’s multiple comparisons test was performed to analyze the log_2_ fold-change of gene expression after Q-PCR. For RNA-seq analysis, the read count was adjusted by TMM, then differential expression analysis was performed by using the EdgeR R package. For microbiome analysis, the classification results were filtered through several statistical post-processing steps (K-mer-based classification, artifact filtering, and species-level abundance estimation [25,26]) to eliminate false-positive results caused by contamination or sequencing artifacts. 

## 3. Results

### 3.1. Mice Survival after Infection with C. difficile

**Figure 1:**
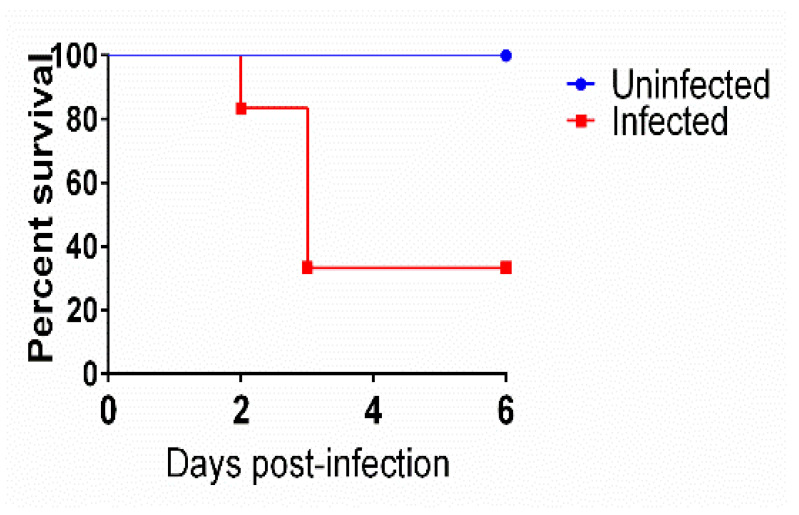
Kaplan–Meier survival curve of mice infected with *C. difficile* ATCC 43255 and uninfected mice.

Antibiotic-treated mice were inoculated with *C. difficile* spores to induce CDI symptoms. As depicted in Figure 1, all uninfected mice survived until the end of the study. On the contrary, 66.6% (4/6) of the infected mice showed severe symptoms of CDI and were euthanized. Interestingly, 33.3% (2/6) of the infected mice showed mild to no CDI-related symptoms and were normal until the end of the experiment. These results are in accordance with previous reports by us and others [18,19,20,21,22].

### 3.2. Transcriptomic Analysis of Symptomatic, Asymptomatic, and Uninfected Mice

#### 3.2.1. RNA-seq Analysis

**Figure 2:**
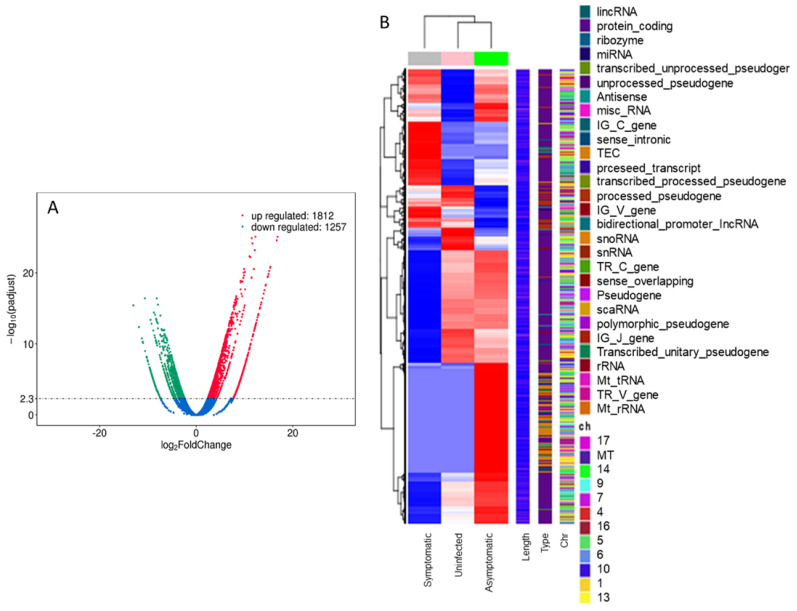
(**A**) Volcano plot (RNA-seq); symptomatic CDI mice had 1812 upregulated and 1257 downregulated genes compared with uninfected mice. (**B**) Overall cluster analysis of differential expression between *C. difficile*-infected symptomatic, asymptomatic mice, and uninfected mice.

Differential expression analysis of the total RNA extracted from the mice cecal mucosa showed 1812 upregulated and 1257 downregulated genes (Figure 2A) when a symptomatic CDI mouse was compared with an uninfected mouse. Clustering of the differentially expressed genes revealed certain gene clusters that were upregulated in the symptomatic mice when compared with uninfected ones. Interestingly, not all these clusters were upregulated in asymptomatic mice. Indeed, asymptomatic mice showed similarity to symptomatic mice in certain gene clusters and similarity to uninfected mice in others (Figure 2B). Out of the upregulated genes in mice with CDI, oncostatin-M *(OSM*), matrix metalloprotease 8 *(MMP8),* triggering receptor expressed on myeloid cells 1 *(Trem-1),* and dual oxidase 2 *(Duox2)* were upregulated several folds (Table 1) and were particularly interesting due to their role in other gastrointestinal conditions [17,27,28,29,30]. We selected these four genes, in addition to TNF-α, to investigate further and confirm their upregulation in CDI via Q-PCR. The RNA-seq-based upregulation of these genes in symptomatic mice relative to the asymptomatic or uninfected ones is shown in Table 1. Other genes that were also upregulated in CDI symptomatic mice included inflammatory cytokines such as CXC motif chemokine ligand 2 (*Cxcl2*, 18.7 log_2_ folds) and 3 (*Cxcl3*, 17.3 log_2_ folds), C-C motif chemokine 3 (*Ccl3*, 16.8 log_2_ folds) and 4 (*Ccl4*, log_2_ 14.9), interleukin 1 alpha and beta (*Il1α and Il1β*, 9.7 and 9.9 log_2_ folds, respectively), and interleukin 1 family member 9 (*Il1f9*, 13.9 log_2_ folds). The upregulated genes also included antimicrobial peptide genes (such as lipocalin 2 [*Lcn2*, 18 log_2_ fold], cathelicidin [*Ngb*, 17.7 log_2_ folds], and calgranulin B [*S100a9*, 17.5 log_2_ folds]) and cytokine receptors (such as interleukin 1 receptor type 2 [*Il1r2*, 10.11 log_2_ folds] and CXC motif chemokine receptor 2 [*Cxcr2*, 9.5 log_2_ folds]). Additionally, certain hypothetical genes were upregulated in CDI mice which we are interested to study their function in the future.

#### 3.2.2. Quantitative PCR

**Figure 3:**
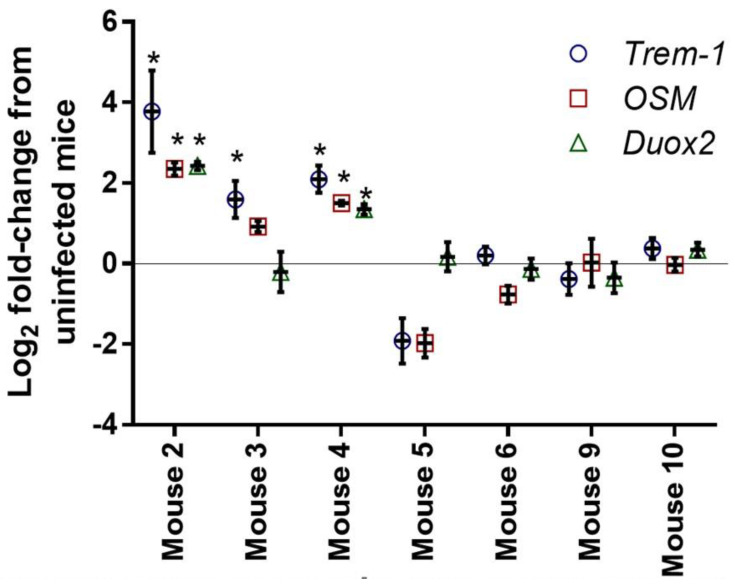
Quantitative PCR (Q-PCR) confirmation of the upregulation of *Trem-1*, *OSM,* and *Duox2* in infected symptomatic mice (2, 3, and 4). No upregulation is observed with asymptomatic (5 and 6) or uninfected (9 and 10) mice. (*) denotes significant difference from the control (uninfected) average using one-way analysis of variance (ANOVA) followed by Tukey’s multiple comparisons test.

We confirmed that the upregulation of the select genes (*OSM*, *Trem-1*, *Duox2*) in the extracted RNA form multiple mice in each group (symptomatic, asymptomatic, and uninfected). As shown in Figure 3, infected symptomatic mice (2, 3, and 4) had a significantly enhanced expression of *OSM*, *Trem-1,* and *Duox2* relative to uninfected mice (9 and 10), while infected asymptomatic mice (5 and 6) did not show this upregulation when compared with uninfected mice. 

### 3.3. Histopathological Analysis of Mice Tissues

**Figure 4:**
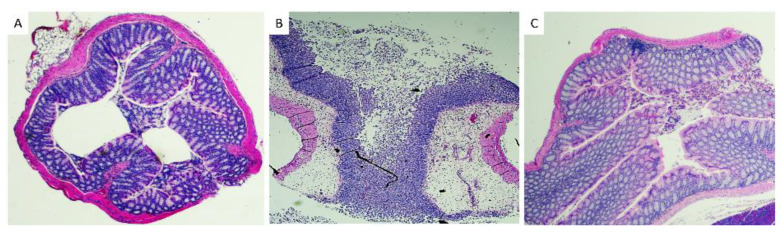
Histological examination of H&E-stained colons of (**A**) uninfected, (**B**) infected symptomatic, and (**C**) infected asymptomatic mice. (**A**,**C**) show normal microscopic morphology, (**B**) shows severe submucosal edema, marked mucosal and luminal neutrophilic infiltrates, and moderate epithelial necrosis and ulceration.

Colon sections were collected from euthanized mice, fixed and stained with H&E to evaluate the inflammatory condition of the mice intestine. Uninfected mice had normal intestinal tissue morphology, whereas symptomatic mice had extensive edema, neutrophilic inflammation, necrosis, and ulceration (Figure 4). Interestingly, infected asymptomatic mice had normal tissue morphology comparable to that of the uninfected mice.

### 3.4. Difference in Microbiome Composition of Infected Symptomatic and Asymptomatic Mice

**Figure 5:**
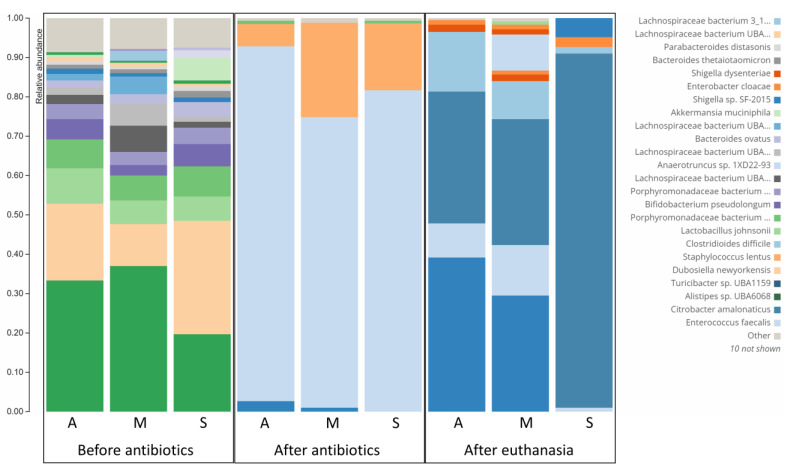
Microbiome analysis of asymptomatic (A), moderately symptomatic (M), and severely symptomatic (S) mice after infection with *C. difficile* spores. Samples were taken from each mouse at three timepoints: before antibiotic administration, after antibiotic administration and before infection, and right before euthanasia.

Data depicted in Figure 5 shows the great reduction in bacterial diversity after administration of antibiotics as reported previously [30]. More importantly, it shows the presence of *C. difficile* in the three mice regardless of their clinical picture. Remarkably, the relative abundance of *C. difficile* was higher in asymptomatic mice and mice with moderate symptoms (15.24% and 9.51%, respectively) than in severely symptomatic mice (1.62%). This is in accordance with previous reports of the same findings where *C. difficile* abundance was not correlated to disease severity [30]. Furthermore, certain bacterial species had interesting patterns of relative abundance in mice with different responses to CDI. After infection with *C. difficile*, the *Turicibacter* species was most abundant in the asymptomatic mouse (42.36%), followed by the moderately symptomatic mouse (22.94%), while it was absent in the severely symptomatic mouse. Interestingly, this bacterial species had a similar pattern in the mice before infection with *C. difficile*. *Turicibacter* species relative abundances were 3.56, 1.17, and 0%, respectively, in asymptomatic, moderately symptomatic, and severely symptomatic mice before infection. This can help in deciphering its controversial role in CDI development [27,28]. The most abundant bacterial strain in the severely symptomatic mouse was *Citrobacter amalonaticus* (87.95%); however, this bacterium comprised 30.24% and 20.79% of the bacterial flora of asymptomatic and moderately symptomatic mice. *Shigella dysentery* was also more abundant in the severely symptomatic mouse (8.77%) than in both asymptomatic and moderately symptomatic mice (3.02 and 1.97%, respectively).

## 4. Discussion

*Clostridioides difficile* infection (CDI) is the most concerning nosocomial infection in the U.S [2]. As per the US Centers for Disease Control and Prevention (CDC), immediate action is required to mitigate the threat imposed by CDI to the public [1]. In 2017 (latest CDC report), CDI caused 223,900 hospitalizations, 12,800 deaths, and over $1 billion in health-care costs within the United States alone [1,2]. CDI symptoms range from asymptomatic to mild to moderate diarrhea. Further, CDI can result in a devastating disease with severe diarrhea, pseudomembranous and fulminant colitis, toxic megacolon, sepsis, and death [29]. *C. difficile* colonizes the colon of patients with imbalanced bacterial flora, usually following antibiotic therapy. Vegetative *C. difficile* cells then release several toxins and mediators to initiate CDI. Importantly, both the host immune system and the pathogen virulence determinants participate in the development and progression of CDI. 

There is conflicting evidence regarding the role of inflammation in CDI progression and the utility of anti-inflammatory drugs in the management of the disease. The usage of anti-inflammatory drugs has been considered a risk factor for developing severe CDI in several studies. These drugs include corticosteroids, NSAIDs (non-steroidal anti-inflammatory drugs, prostaglandin inhibitor), anti-TNF-α monoclonal antibodies (mAbs), and histamine receptor blockers [14,15,16,17,31,32,33,34,35]. Pretreating mice with indomethacin (an NSAID) or anti-TNF-α mAb before infecting with *C. difficile* resulted in more severe CDI [36,37,38]. Conversely, anti-inflammatory drugs were reportedly beneficial in controlling the symptoms of CDI and reducing the febrile response in animal models [33,34]. In humans, the addition of anti-inflammatory agents helped to manage severe CDI cases that were refractory to treatment with standard anticlostridial antibiotics [31,35,39,40]. In several other studies, there was no correlation between the anti-inflammatory drugs and the development or severity of CDI [32,41,42].

In the current study, we utilized the observation from many previous studies, including ours, that a subpopulation of infected mice remains clinically normal [18,19,20,21,22] (Figure 1). Histological examination showed mice with symptomatic CDI to have severe intestinal inflammation, edema, and mucosal necrosis. However, clinically normal *C. difficile*-infected mice have normal intestines in spite of being *C. difficile* positive in both transcriptomic and microbiome analyses of cecal contents (Figure 3, Figure 4 and Figure 5).

We utilized RNA-seq and Q-PCR to identify genes that are upregulated in symptomatic CDI mice. Out of the large number of upregulated genes, we selected five proinflammatory genes that were either not upregulated or slightly upregulated in asymptomatic infected mice (Table 1). These genes are oncostatin-M (*OSM*), matrix metalloprotease 8 (*MMP8*), triggering receptor expressed on myeloid cells 1 (*Trem-1*), dual oxidase 2 (*Duox2*), and tumor necrosis factor alpha (*TNF-α*).

OSM and TNF-α are inflammatory cytokines elevated in CDI and irritable bowel syndrome (IBD) [17]. Anti-TNF-α mAb (infliximab) is approved for the management of IBD, and anti oncostatin-M (GSK2330811) is currently being evaluated in clinical trials for controlling IBD [34,37,39,43,44]. MMPs have been found to provide a nutritional source for *C. difficile* during inflammation and to exclude opposing members of the normal gut flora [17]. Inhibition of MMPs was generally found beneficial in controlling intestinal and other inflammations in a number of animal models [45]. Trem-1 is expressed by neutrophils and is involved in mediating and amplifying inflammatory processes. Trem-1 is also thought to be a potential target for the management of IBD. Inhibition of Trem-1 activity (either through genetic mutation or by the use of inhibitor peptides) restored normal functions and prevented inflammation in experimentally-induced colitis in mice [46,47]. Duox2 is a NADPH oxidase family protein that is capable of releasing hydrogen peroxide. It was shown to play a role in active inflammation during ulcerative colitis [48]. Identifying these genes as important players in CDI-induced inflammation raises the question whether inhibiting these proteins could reduce CDI severity. 

Microbiome analysis study further confirmed the presence of *C. difficile* in the colonic content of severely symptomatic, moderately symptomatic, and asymptomatic CDI mice. Interestingly, the relative abundance of *C. difficile* in the colonic content was not correlated to the severity of the disease. Additionally, the abundance of *Turicibacter* species either before or after the infection with *C. difficile* was inversely correlated to the severity of the disease. This indicates the potential role of this bacteria in the protection against [28,29] CDI and warrants further investigation to be used as a treatment of CDI or at least as a predictor of the disease severity. On the other hand, certain bacterial species were enriched in severe CDI (e.g., *Citrobacter amalonaticus* and *Shigella dysentery*).

In conclusion, this is a small-scale differential transcriptomics and microbiome study of CDI at different severities in mice. The study sheds the light on the potential role of certain inflammatory pathways in CDI severity. This, in turn, opens the door for the use of new classes of anti-inflammatory treatments for CDI. In addition, this study correlated the CDI status to the microbiome composition and pinpoints certain bacterial strains that potentially participate in CDI severity either positively or negatively. Although the study is limited by the small number of mice used, it warrants further investigation to confirm its primary findings.

## Figures and Tables

**Table 1:** Log_2_ fold-change of selected genes between infected symptomatic mice when compared with uninfected or infected asymptomatic mice.

Gene ID	OSM ^1^	MMP8 ^2^	Trem-1 ^3^	Duox2 ^4^	TNF-α ^5^
Infected symptomatic versus uninfected	11.0	15.3	11.9	9.8	7.3
Infected symptomatic versus infected asymptomatic	8.5	9.7	6.8	3.3	4.6

^1^ Oncostatin-M, ^2^ Matrix metalloproteinase 8, ^3^ Triggering receptor expressed on myeloid cells 1, ^4^ Dual oxidase 2, ^5^ Tumor necrosis factor-α.

## Data Availability

Not applicable.
